# Editorial: Machine learning methods in single-cell immune and drug response prediction

**DOI:** 10.3389/fgene.2023.1233078

**Published:** 2023-06-19

**Authors:** Ren Qi, Quan Zou

**Affiliations:** ^1^ School of Life Science and Technology, University of Electronic Science and Technology of China, Chengdu, China; ^2^ Yangtze Delta Region Institute (Quzhou), University of Electronic Science and Technology of China, Quzhou, China; ^3^ Institute of Fundamental and Frontier Sciences, University of Electronic Science and Technology of China, Chengdu, China

**Keywords:** single-cell sequencing data, single-cell immune, drug response prediction, machine learning, feature selection

Machine learning methods play a crucial role in single-cell immune and drug response prediction. This field of research aims to understand the heterogeneity and complexity of the immune system at the single-cell level and how it interacts with drugs or therapies. By leveraging machine learning techniques, scientists can extract meaningful insights from high-dimensional and large-scale single-cell data, leading to improved understanding and prediction of immune responses to drugs.

Given that single-cell sequencing is suited to investigating cell-type-specific patterns of response to therapy, this Research Topic explores the potential for machine learning applied to characterize the single-cell transcriptomes of cancer and immune cells. We are pleased to accept the following five papers with the new sight of machine learning techniques selected from all submissions.

Machine learning methods facilitate the integration of single-cell data with other omics data types, such as bulk RNA-seq, proteomics, or epigenomics. To identify cell types in complex tissues and integrate reference-bulk RNA sequencing data and single-cell transcriptomes, Liu et al. present a linear fast semi-supervised clustering model. Integrative analysis can uncover multi-level relationships, identify regulatory mechanisms, and provide a more comprehensive view of immune responses and drug actions.

Two papers concentrate on cell type identification in this Research Topic. Cell-type identification involves the classification and annotation of individual cells into distinct cell types or cell states based on their gene expression profiles. It is crucial for understanding cellular heterogeneity, uncovering developmental processes, discovering novel cell types, linking cell types to functions, enabling comparative analyses, and integrating data across multiple modalities in scRNA-seq data analysis. Song et al. identified cell types for scRNA-seq based on a deep-learning transformer. Their innovative use of transformer annotation cells not only improves accuracy, but also forms the foundation for further downstream analyses and interpretation, ultimately leading to a deeper understanding of cellular biology, disease mechanisms, and therapeutic strategies. In addition, Li et al. employed a progressive clustering method to identify cell populations in scRNA-seq data, they described data with clustering trees and revealed the structure of both abundant cell populations and rare cell populations. Single-cell data can capture dynamic changes in cellular states during immune responses or drug treatments. Meanwhile, machine learning methods enable the characterization of developmental processes, lineage relationships, and how cells respond to perturbations, providing insights into immune system dynamics and drug responses. These models can then be used to guide drug development, personalize therapies, or uncover novel therapeutic targets.

Moreover, two papers accomplished classification tasks by traditional machine learning algorithms. For example, Xu et al. proposed a classification method for the characterization of chromatin accessibility patterns at single-cell resolution. Chromatin accessibility refers to the physical accessibility of DNA sequences within the chromatin structure. Changes in chromatin accessibility play a fundamental role in various biological processes, including development, differentiation, and response to environmental cues. Xu’s method combined Monte Carlo feature selection with incremental feature selection, yielding essential genes, classification rules, and an efficient random forest (RF) classifier. Besides, Li et al. constructed an optimal classifier with a decision tree and RF to study immune responses to COVID-19 vaccination strategies and analyze scRNA-seq data from multiple tissues. These methods provided insights into gene regulation, development, and disease mechanisms, and can aid in the development of novel therapeutic approaches.

In summary, machine learning methods enable the extraction of valuable insights from high-dimensional single-cell data, facilitating a deeper understanding of immune responses and the prediction of drug effects. Papers in this Research Topic have the potential to promote the development of precision medicine and advance our understanding of the immune system. Finally, we thank all efforts of the authors, reviewers, and staff at the Frontiers in Genetics editorial office.

